# General practitioners’ awareness of depressive symptomatology is not associated with quality of life in heart failure patients – cross-sectional results of the observational RECODE-HF Study

**DOI:** 10.1186/s12875-017-0670-9

**Published:** 2017-12-08

**Authors:** Marion Eisele, Sigrid Boczor, Anja Rakebrandt, Eva Blozik, Jens-Martin Träder, Stefan Störk, Christoph Herrmann-Lingen, Martin Scherer, for the RECODE-HF Study Group, Winfried Adam, Cassandra Behrens, Eva Blozik, Sigrid Boczor, Marion Eisele, Malte Harder, Christoph Herrmann-Lingen, Agata Kazek, Dagmar Lühmann, Anja Rakebrandt, Koosje Roeper, Martin Scherer, Stefan Störk, Jens-Martin Träder

**Affiliations:** 1 Department of Primary Medical Care, Center for Psychosocial Medicine, University Medical Center Hamburg-Eppendorf, Martinistraße 52, 20246 Hamburg, Germany; 2 Department of Primary Medical Care, University of Luebeck, Ratzeburger Allee 160, 23538 Luebeck, Germany; 3 Comprehensive Heart Failure Center Würzburg, University and University Hospital Würzburg, Straubmühlweg 2a, 97078 Würzburg, Germany; 4 University of Göttingen Medical Center, and German Center for Cardiovascular Research, partner site Göttingen, von-Siebold-Str. 5, D-37099 Göttingen, Germany

**Keywords:** Depression, Heart failure, Recognition of depression, Quality of life, Depression treatment, Observational study, Primary care, Healthcare research, Depressive symptomatology

## Abstract

**Background:**

Depression is a common comorbidity in patients with chronic heart failure (HF) and linked to a wider range of symptoms which, in turn, are linked to a decreased health-related quality of life (HRQOL). Treatment of depression might improve HRQOL but detecting depression is difficult due to the symptom overlap between HF and depression. Therefore, clinical guidelines recommend to routinely screen for depression in HF patients. No studies have so far investigated the treatment after getting aware of a depressive symptomatology and its correlation with HRQOL in primary care HF patients. Therefore, we examined the factors linked to depression treatment and those linked to HRQOL in HF patients. We hypothesized that GPs’ awareness of depressive symptomatology was associated with depression treatment and HRQOL in HF patients.

**Methods:**

For this observational study, HF patients were recruited in primary care practices and filled out a questionnaire including PHQ-9 and HADS. A total of 574 patients screened positive for depressive symptomatology. Their GPs were interviewed by phone regarding the patients’ comorbidities and potential depression treatment. Descriptive and regression analysis were performed.

**Results:**

GPs reported various types of depression treatments (including dialogue/counselling by the GP him/herself in 31.8% of the patients). The reported rates differed considerably between GP-reported initiated treatment and patient-reported utilised treatment regarding psychotherapy (16.4% vs. 9.5%) and pharmacotherapy (61.2% vs. 30.3%). The GPs' awareness of depressive symptomatology was significantly associated with the likelihood of receiving pharmacotherapy (OR 2.8; *p* < 0.001) but not psychotherapy. The patient’s HRQOL was not significantly associated with the GPs' awareness of depression.

**Conclusion:**

GPs should be aware of the gap between GP-initiated and patient-utilised depression treatments in patients with chronic HF, which might lead to an undersupply of depression treatment. It remains to be investigated why GPs’ awareness of depressive symptomatology is not linked to patients’ HRQOL. We hypothesize that GPs are aware of cases with reduced HRQOL (which improves under depression treatment) and unaware of cases whose depression do not significantly impair HRQOL, resulting in comparable levels of HRQOL in both groups. This hypothesis needs to be further investigated.

## Background

About 61.7 billion people worldwide suffer from chronic heart failure (HF) [1], a disease with high rates of mortality and hospital admissions [2, 3]. Depression rates have been found to be higher in HF patients than in patients without HF [4–6] and have been independently predictive of poor clinical outcomes [7]. Furthermore, depression in HF patients has been linked to a wider range of symptoms, which in turn have been linked to a decreased quality of life [8]. Therefore, treatment of depression may significantly improve patients’ quality of life, one of the treatment goals specified in the clinical guidelines for the diagnosis and treatment of heart failure [9, 10].

At the same time, detecting depression is particularly difficult due to the symptom overlap between HF and depression. Earlier studies found recognition rates of depression between 57.5% [11] and 63.2% [12] in hospitalised HF patients. The authors investigated to what extent general practitioners (GPs) were aware of their HF patients’ depressive symptomatology and found a rate of 35.0%–50.7% (depending on the definition of awareness) [13]. Deficient awareness of depressive symptomatology in HF patients might result in undersupply of depression treatment and reduced quality of life in HF patients. Crystal et al. found that patients with heart disease received significantly less depression treatment in terms of psycho- or pharmacotherapy [14] than patients without heart disease.

Therefore, national and international clinical guidelines recommend to routinely screen patients with HF for depression [10, 15–18]. For a number of years, studies have been investigating if the treatment of depression may also have an effect on HF prognosis, but so far there has not been a definite answer to this question. Interventional trials have found that the pharmacological treatment of depression in HF patients does not reduce the severity of depression nor does it have a positive effect on HF outcomes [19–21]. Conversely, cognitive behavioural therapy has been found to improve depression as well as quality of life [22]. However, so far, no studies have investigated the therapeutic procedures after getting aware of a depressive symptomatology and its potential link to patients’ health related quality of life (HRQOL) in primary care HF patients.

Therefore, the study objectives were: (1) To describe how GPs treat depressive symptomatology in HF patients. (2) To describe the congruence between GP-initiated and patient-utilised depression treatment. (3) To examine the factors associated with depression treatment in HF patients. (4) To examine the factors associated with HRQOL of HF patients with depressive symptomatology. We hypothesized that the GPs’ awareness of depressive symptomatology is significantly associated with a higher utilisation of pharmacotherapy and psychotherapy. We further hypothesized that the GPs’ awareness of depressive symptomatology was significantly associated with a higher HRQOL.

## Methods

In this observational study, primary care surgeries and their HF patients were recruited in Germany between 2/2012 and 6/2014. Baseline data were collected between 8/2012 and 11/2014. A detailed description of all study procedures has been described elsewhere [13, 23]. To summarise: All GPs in four German cities and surrounding areas received a written invitation to participate in the study and were contacted by phone, if they had not responded to the invitation letter. Primary care HF patients were recruited by their GP. Those patients who agreed to participate returned the informed consent letters to the study centre and in return received a baseline questionnaire by mail. Inclusion criteria were an age of 18 years or older, a diagnosis of chronic HF documented within the last 5 years, and at least one GP contact within the last 6 months. Exclusion criteria were dementia, death since the last GP visit and HF patients who were not regular patients of the participating GP practice.

Patient questionnaires contained questions regarding patients’ psychosocial comorbidity (using the Hospital Anxiety and Depression Scale (HADS) including the anxiety (HADS-A) and depression (HADS-D) subscales [24, 25], Patient Health Questionnaire 9-item depression subscale (PHQ-9) [26–28], as well as selected items from the PROMIS depression and anxiety scales [29, 30]), HRQOL (using the EQ-5D visual analogue scale (EQ-5D VAS) [31]), use of psychotherapy (any psychotherapy, not restricted to those approved by health insurance companies), and current medications taken to treat depression, anxiety, agitation, sleep disturbance or “burnout”. Drug names were verified whether they classified as prescription drugs and were then converted to ATC codes. If an ATC code included antidepressant ingredients and the drug was a prescription drug, the drug was coded as “prescription antidepressant”.

The questionnaires were analysed by a validated algorithm [13], established to identify depression in patients with HF. All patients with valid criteria 2 of the hierarchical algorithm (PHQ > 8 and HADS-D > 8) were selected. Due to the positive predictive value of 68.8% for a depression diagnosis [13], this included patients with depression and patients with depressive symptoms, later referred to as P(+) patients (patients with depressive symptomatology). A telephone interview was conducted with each GP of the P(+) patients identified by the algorithm regarding the patients’ somatic and psychological comorbidities. At the beginning of the interview, the GP was asked to access the patient records in the computer, in order to be able to use all data available (patient record as well as recollections). All questions were answered for each patient separately. If the GP answered “Yes” to the question: “Does the patient currently display depressive symptomatology?”, he/she was asked to differentiate whether he/she thought the symptoms indicated a clinical depression or sub-threshold symptoms and what types of measures he had taken to treat said symptoms (“Which treatment did you initiate?”). Of all comorbidities stated by the GP, the numbers of all comorbidities indexed in the MultiCare comorbidity list of chronic conditions [32] were summed up for the comorbidity score.

### Patient groups for analyses

**Fig. 1:**
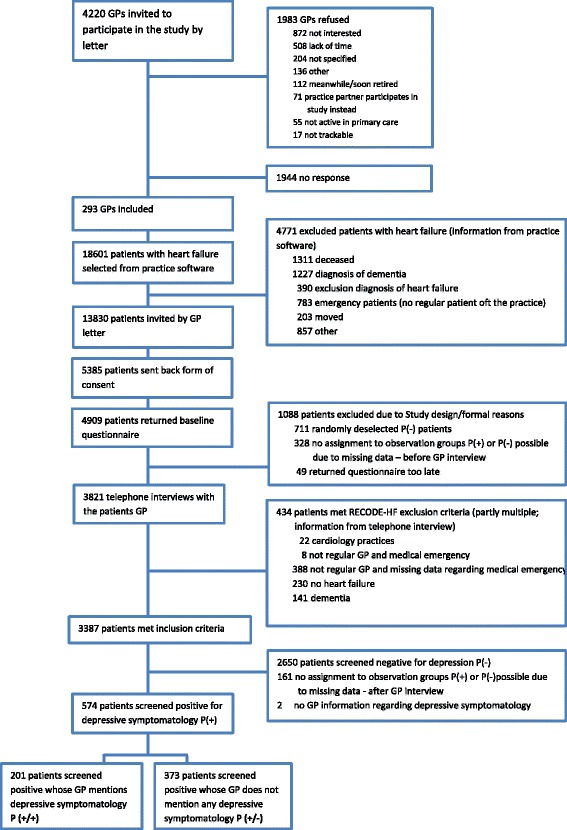
Sampling frame

Depending on the information given in the GP interview, patients were grouped for analysis (see Fig. 1). P(+) patients, whose GP stated that they currently displayed depressive symptoms or depressive disorder were assigned as P(+/+). P(+) patients, whose GP stated that they currently did not display depressive symptoms or depressive disorder, were assigned as P(+/−).

### Statistical analyses

The baseline data were analysed using SPSS Version 20 and 22. Descriptive analyses were performed to address study objectives (1) and (2). To compare the subgroups of P(+/+) and P(+/−) patients, t-tests and chi-squared tests were conducted to test for significant differences where appropriate. Logistic regression models were conducted to examine the factors associated with depression treatment in patients with HF (study objective 3). Besides age, gender, and education, NYHA Class and depression scale PHQ-9 were included as potential confounders to check for disease severity of the study population. Three univariate ANCOVAs were carried out to examine the factors associated with HRQOL of HF patients with depression (study objective 4). Three models with different independent factors were examined: the GP’s awareness of depressive symptomatology (model 1), the depression treatment (model 2), and the GP’s awareness of depressive symptomatology, depression treatment,and the interaction between both (model 3). All three models were controlled for the patient’s age, gender and education. Furthermore - due to their link to HRQOL in HF patients - NYHA Class and severity of depression (PHQ-9 Score) [33] as well as the number of comorbidities [34] were included as potential confounders. The three models were tested for a GP cluster effect. The nominal level of significance was set at *p* = 0.05. No data imputation strategies were applied.

### Ethics approval

The study was conducted in compliance with the Declaration of Helsinki. The study protocol was approved by the local ethics committees (Medical Association of Hamburg, Approval No. PV3889; Ethics Committee of the Medical Faculty of the University of Würzburg, Approval No. 125/12, Ethics Committee of the University of Göttingen Medical Center, Approval No. 19/8/11).

## Results

### Sampling frame

Of 13,830 HF patients invited by their GPs to join the study, 4909 patients consented to participate in the study and returned a completed baseline questionnaire. Of all 4909 incoming questionnaires, 690 (14.1%) screened positive for depressive symptomatology P(+). Of those, 574 patients from 224 GPs were eligible for further analysis (see Fig. 1). On average, each GP referred 2.6 (SD 1.8; range 1–12) patients.

The GPs of 201 of the 574 patients (35.0%) were aware of their patients’ depressive symptomatology P(+/+). Patient and GP characteristics are displayed in Tables 1 and 2.

**Table 1:** Patient characteristics

Characteristics	Patients screened positive for depressive symptomatology			Total valid *n*	Test of significant differences
	GP aware of depressive symptomatology	GP unaware of depressive symptomatology	Total		
	P (+/+) (*n* = 201)	P (+/−) (*n* = 373)	P (+) (*n* = 574)		
Age, mean (SD)	71.9 (11.7)	73.3 (11.4)	72.8 (11.5)	540	*p* = 0.168
Male gender, N (%)	88 (43.8%)	190 (50.9%)	278 (48.4%)	562	*p* = 0.066
Education level (CASMIN 3)				562	*p* = 0.020
Low	131 (65.2%)	280 (75.1%)	411 (71.6%)		
Middle	51 (25.4%)	67 (18.0%)	118 (20.6%)		
High	16 (8.0%)	17 (4.6%)	33 (5.7%)		
NYHA classification, *N* (%)				566	*p* = 0.218
Class I	30 (14.9%)	60 (16.1%)	90 (15.7%)		
Class II	80 (39.8%)	180 (48.3%)	260 (45.3%)		
Class III	74 (36.8%)	110 (29.5%)	184 (32.1%)		
Class IV	12 (6.0%)	20 (5.4%)	32 (5.6%)		
Comorbidity score, mean (SD)	4.9 (2.5)	5.1 (2.6)	5.0 (2.5)	563	*p* = 0.494
PHQ-9 Score, mean (SD)	14.3 (4.4)	13.0 (3.5)	13.4 (3.9)	574	*p* < 0.001
Health-related quality of life (EQ-5D VAS), mean (SD)	38.2 (19.2)	41.7 (17.6)	40.5 (18.3)	558	*p* = 0.029

**Table 2:** GP characteristics

GP characteristics		Total valid *n*
Years of GP practice, mean (SD)	15.4 (9.5)	224
Male, *N* (%)	167 (74.6)	217
Type of practice, *N* (%)		223
Single practice	108 (48.4)	
Shared practice	102(45.7)	
Group practice	13 (5.8)	
Any psychological specialisation, *N* (%)	43 (19.2)	224
Medical specialist training in psychiatry, neurology or psychosomatics	4 (1.8)	224
Additional training in psychotherapy	41 (18.3)	224

#### How do general practitioners treat depressive symptomatology in heart failure patients?

##### Pharmacological and psychological treatment (patient information) stratified by GPs' awareness of depressive symptomatology

Regarding all P(+) patients, 6.4% indicated that they utilised psychotherapy, 18.6% reported taking prescription antidepressants and a total of 22.3% utilised psychotherapy, pharmacotherapy or both. All rates were significantly higher in P(+/+) patients than in P(+/−) patients (see Table 3).

**Table 3:** Patients' indications regarding utilisation of prescription antidepressants and psychotherapy

	P (+/+)	P (+/−)	Total P (+)	Test of significant differences
	N (%)	N (%)	N (%)	
Psychotherapy	19 (9.5%)	18 (4.8%)	37 (6.4%)	*p* = 0.021
Taking any prescription antidepressant	61 (30.3%)	46 (12.3%)	107 (18.6%)	*p* < 0.001
Antidepressants and/or psychotherapy	71 (35.3%)	57 (15.3%)	128 (22.3%)	*p* < 0.001
N	201 (100%)	373 (100%)	574 (100%)	

Comparison of patients whose GPs were aware (P(+/+)) of their patients’ depressive symptomatology and those whose GPs were unaware (P(+/−)). Compared were cases with utilisation versus all others (no utilisation and cases with no available information, respectively). Absolute and relative frequencies were given

##### Measures taken if GP was aware of depressive symptomatology (GP information)

The measures taken if GPs were aware of their patients' depressive symptomatology are displayed in Table 4 for all P(+/+) patients and divided depending on whether GPs stated depressive symptoms or depressive disorder in their patients. No information is displayed for P(+/−) patients, because GPs were not asked about measures taken if no depressive symptoms or depressive disorder were indicated by GPs. GPs prescribed pharmacotherapy to 75.8% of their patients, whom they judged as having a depressive disorder and referred 42.4% to a specialist. Only 5.3% of the patients judged by their GPs as having a depressive disorder did not receive treatment. If GPs stated that the patients currently displayed depressive symptoms, they still initiated pharmacotherapy in 33.3% of the cases and referred 10.1% to specialists. GPs most often (in 43.5% of the cases) offered this group of patients consultation/counselling carried out by themselves (see Table 4).

**Table 4:** GPs indications regarding measures taken by GPs if aware of depressive symptomatology (P(+/+); *N* = 201)

	GP measures if aware of depressive symptomatology P(+/+)		
Measures	GP judgement: depressive symptoms	GP judgement: depressive disorder	Total
Pharmacotherapy	23 (33.3%)	100 (75.8%)	123 (61.2%)
Consultation/Counselling by GP	30 (43.5%)	34 (25.8%)	64 (31.8%)
Referral to specialists	7 (10.1%)	56 (42.4%)	63 (31.3%)
Psychotherapist	5 (7.2%)	28 (21.2%)	33 (16.4%)
Neurologist	1 (1.4%)	18 (13.6%)	19 (9.5%)
Psychiatrist	1 (1.4%)	18 (13.6%)	19 (9.5%)
Other specialist	0 (0%)	1 (0.8%)	1 (0.5%)
Inpatient treatment	0 (0%)	6 (4.5%)	6 (3.0%)
Counselling by others than GP (eg, counselling centre)	0 (0%)	1 (0.8%)	1 (0.5%)
Other	5 (7.2%)	6 (4.5%)	11 (5.5%)
Pain therapy	2 (2.9%)	1 (0.8%)	3 (1.5%)
Physiotherapy/Rehabilitation	1 (1.4%)	2 (1.5%)	3 (1.5%)
Psychosomatic care	1 (1.4%)	1 (0.8%)	2 (1.0%)
Patient rejects treatment	0 (0%)	2 (1.5%)	2 (1.0%)
Active watchful waiting	2 (2.9%)	0 (0%)	2 (1.0%)
No treatment initiated	14 (20.3%)	7 (5.3%)	21 (10.4%)
*N*	69 (100%)	132 (100%)	201 (100%)

Absolute and relative frequencies were given

Regarding the 123 *P* (+/+) patients whose GPs stated that they had initiated pharmacotherapy, 72 patients (58.5%) did not state that they took any prescription antidepressants. Of those 72 patients, 36.1% received counselling/consultations from their GPs, 23.6% were referred to specialists by their GPs, 2.8% received hospital admission referrals and 4.2% different types of treatment. In total, 40 of the 72 patients (55.6%) received other types of treatment.

#### Factors associated with depression treatment in heart failure patients

The association between awareness of depressive symptomatology and depression treatment was investigated using two logistic regression models investigating the endpoints: self-reported utilisation of psychotherapy (see Table 5) and of self-reported utilisation of prescription antidepressants (see Table 6). While GPs' awareness of depressive symptomatology was not significantly associated with the probability of self-reported utilization of psychotherapy, older age lowered the chance to utilise psychotherapy. GP’s awareness of depressive symptomatology as well as higher PHQ-9 score were both associated with self-reported utilisation of prescription antidepressants.

**Table 5:** Association between GPs' awareness of depressive symptomatology and self-reported utilization of psychotherapy in all P(+) patients (logistic regression)

Variable	*P*-Value	Odds Ratio [95% CI]
GPs' awareness of depressive symptomatology (reference: unawareness)	0.081	2.011 [0.919; 4.401]
Education level (reference: Primary education)	0.506	
Secondary education	0.243	1.628 [0.718; 3.688]
Tertiary education	0.821	1.206 [0.238; 6.127]
Age	<0.001	0.926 [0.894; 0.958]
Gender (reference: male)	0.722	1.153 [0.527; 2.522]
NYHA Class (reference: NYHA Class 1)	0.621	
NYHA Class 2	0.273	0.593 [0.233; 1.510]
NYHA Class 3	0.289	0.556 [0.188; 1.645]
NYHA Class 4	0.376	0.366 [0.040; 3.376]
PHQ-9 Score	0.876	1.007 [0.923; 1.099]
Constant	0.059	14.342

*N* = 413; Nagelkerkes R^2^ = 0.197

**Table 6:** Association between GPs' awareness of depressive symptomatology and self-reported utilization of prescription antidepressants in all P(+) patients (logistic regression)

Variable	*P*-Value	Odds Ratio [95% CI]
GPs' awareness of depressive symptomatology (reference: unawareness)	<0.001	2.845 [1.758; 4.604]
Education level (reference: Primary education)	0.670	
Secondary education	0.372	0.765 [0.425;1.377]
Tertiary education	0.868	0.919 [0.338; 2.499]
Age	0.306	0.989 [0.968; 1.010]
Gender (reference: male)	0.136	1.451 [0.889; 2.367]
NYHA Class (reference: NYHA Class 1)	0.421	
NYHA Class 2	0.545	0.821 [0.433; 1.557]
NYHA Class 3	0.217	0.642 [0.318; 1.297]
NYHA Class 4	0.160	0.408 [0.117; 1.424]
PHQ-9 Score	0.001	1.108 [1.045; 1.174]
Constant	0.012	0.089

*N* = 525; Nagelkerkes *R* ^2^ = 0.141

#### Factors associated with the health-related quality of life of heart failure patients with depressive symptomatology

Table 7 displays the association between patients’ HRQOL and GPs’ awareness of depressive symptomatology (model 1), depression treatment (model 2) and awareness of depressive symptomatology, depression treatment as well as the interaction between both (model 3). GPs' awareness of depressive symptomatology was not significantly associated with HRQOL. As there was no significant GP cluster effect, the GP cluster was excluded from the models.

**Table 7:** Association between GPs' awareness of depressive symptomatology, depression treatment and HRQOL

	Model 1		Model 2		Model 3	
Variable	F	*P*-Value	F	*P*-Value	F	*P*-Value
Corrected model	8.968	<0.001	9.107	<0.001	7.602	<0.001
Constant	96.808	<0.001	98.089	<0.001	97.612	<0.001
GPs' awareness of depressive symptomatology	0.122	0.727			0.436	0.510
Utilisation of prescription antidepressants or psychotherapy			1.303	0.254	1.519	0.218
GPs' awareness of depressive symptomatology* Utilisation of prescription antidepressants or psychotherapy					0.087	0.768
Age	4.021	0.045	3.394	0.066	3.512	0.062
Sex (female)	1.440	0.231	1.747	0.187	1.597	0.207
Education	3.052	0.048	3.057	0.048	3.077	0.047
NYHA Class	10.399	<0.001	10.327	<0.001	9.942	<0.001
Comorbidity score	0.447	0.504	0.285	0.594	0.273	0.601
PHQ-9 Score	33.030	<0.001	35.974	<0.001	34.418	<0.001
	*N* = 503; R^2^ = 0.154		*N* = 503; R^2^ = 0.156		*N* = 503; R^2^ = 0.157	

Univariate ANCOVA with endpoint HRQOL (EQ-5D VAS) in patients screened positive for depressive symptomatology P(+). Regarding utilisation of prescription antidepressants or psychotherapy, cases with utilisation were compared with all other cases (no utilisation and cases with no available information, respectively)

Table 8 displays HRQOL for all P(+) patients depending on GP’s awareness of depressive symptomatology and depression treatment.

**Table 8:** HRQOL depending on depression treatment and GPs' awareness of all P(+) patients

Utilisation of prescription antidepressants or psychotherapy	GP aware of depressive symptomatology	*N*	mean	SD
No utilization or not specified	No	304	41.7	17.5
	Yes	127	37.5	18.6
Yes	No	56	41.5	18.4
	Yes	71	39.3	20.3

## Discussion

### Main results

This observational study revealed that the GPs' awareness of depressive symptomatology was significantly associated with patients’ self-reported utilisation of prescription antidepressants and not to patients’ self-reported utilisation of psychotherapy. Even though GPs stated that they had referred 16.4% of P(+/+) patients to psychotherapists, only 9.5% of those patients reported utilising psychotherapy. The respective numbers of pharmacotherapy are 61.2% GP-initiated versus 30.3% patient-utilised pharmacotherapy. Patients’ HRQOL was not significantly associated with their GPs’ awareness of depressive symptomatology.

### Strengths and limitations

This has been the first study investigating the treatment of depressive symptomatology and its link to GPs' awareness in a large study of 574 primary care HF patients with comorbid depressive symptomatology. The fact that an algorithm was applied to identify patients with depressive symptomatology, controlling the overlapping symptoms of depression and HF, made this study unique. We believe that fewer false positive cases of HF patients with depressive symptomatology have been included in this study than in other studies using instruments with cut-off values for patients without HF.

This study also has certain limitations. Due to the study procedure, a potential selection bias of patients less impaired by HF and/or depression may have occurred, because severely depressed patients may not have been willing to participate in the study. Therefore, the characteristics of the patients included in the analyses are presented in detail (including the mean PHQ-9 value) in Table 1. Furthermore, due to the study design, patients with depressive symptomatology were identified and included in the study. Next, GPs were asked if they believed these patients to actually display a current depressive symptomatology. Patients with depression who were successfully treated (e.g. with pharmacotherapy), were not identified through the algorithm due to decreased depressive symptomatology. Therefore, we cannot rule out that patients responding less favourably to depression treatment are overrepresented in the study. This may lead to a higher rate of patients who do not adhere to the recommended treatment. Additionally, the number of depression treatment is likely to be underestimated in the present study due to the selection of patients with acute depressive symptomatology. Those patients undergoing successful treatment and currently free from symptoms may be underrepresented. Also, our results are based on patients with depressive symptomatology and we have not been able to differentiate between patients with depression diagnosis and patients with a subthreshold symptomatology. If only patients with a diagnosis of depression had been included, the results might have been different. However, even though results of our study are not representative for patients diagnosed with depression, there is evidence that patients with subthreshold depressive symptomatology or symptomatology, which is not considered clinically relevant, also experience a reduced quality of life [35] and even higher morbidity and mortality rates after myocardial infarction [36] .

#### How do general practitioners treat depressive symptomatology in heart failure patients?

##### Treatment depending on the GP’s rating of symptom severity

The initiated treatment of patients whose GP was aware of their depressive symptomatology (P(+/+)), differed depending on the GP’s rating whether a patient displayed “depressive symptoms” or “depressive disorder”. Consistent with national and international guidelines [37–39], GPs initiated more pharmacotherapy (75.8% vs. 33.3%) and referrals to psychotherapists (21.2% vs. 7.2%) when he/she believed that the patient was suffering from “depressive disorder”, than in patients believed to be just displaying “depressive symptoms”. Only in 5.3% of patients believed to suffer from “depressive disorder” did GPs not initiate treatment. The respective percentage was higher in patients rated as just displaying “depressive symptoms” (20.3%). This is consistent with the “active monitoring” approach in mild or sub-threshold depression. Even though several GPs referred to this strategy as “treatment”, we assumed that most GPs did not refer to this strategy as treatment in our open question “Which treatment did you initiate?”. Dialogue or counselling was the most frequent GP-initiated treatment in patients rated as displaying “depressive symptoms” (43.5%). This step-by-step approach corresponds to primary care proceedings and does not indicate any treatment gaps in *P*(+/+) patients.

##### Incongruences in GP-initiated versus patient-utilised treatment

Regarding pharmacotherapy, the reported GP-initiated treatment and the patient-reported treatment utilisation differed considerably in all patients whose GPs were aware of their depressive symptomatology (P(+/+)), (61.2% GP prescription vs. 30.3% patient utilisation) and psychotherapy (16.4% GP referrals to psychotherapists vs. 9.5% patient utilisation of psychotherapy). These differences were in line with prior research focusing on barriers to depression treatment in primary care: The study by Nutting et al. concluded that guideline-appropriate treatment barriers were most frequently caused by patient-centred factors (such as a resistance to the diagnosis, treatment resistance or non-compliance) [40]. In regards to psychotherapy, the authors of the present study assume that a lack of availability may be an additional key factor. Due to social desirability and a likely polypharmacy based on the age of the patients, patients might have significantly underreported their utilisation of pharmaco- and psychotherapy while GPs might have reported more measures than they had actually initiated. However, it should be considered that a remaining gap may still be likely between the initiation and utilisation of pharmacotherapy, when treating depression in HF patients. Besides the above-mentioned patient barriers found by Nutting et al., interaction effects of antidepressants and HF pharmacotherapy as well as cardiac sideeffects of antidepressants as described in detail by Rustad et al. [41] might decrease patient adherence and lead to the findings in the present study.

Another aspect is the question whether the gap between GP-initiated and patient-utilised pharmacotherapy is filled therapeutically by alternative treatment options, such as counselling by the GP or referrals to other specialists. In our data, 55.6% of patients who did not report the utilising pharmacotherapy although their GP had initiated pharmacotherapy, stated that their GP had also initiated an additional type of treatment. Therefore, even those patients who had not utilised the prescribed pharmacotherapy, might have received (appropriate) treatment. This is what Weel et al. refer to as “treating the patient as a whole” in primary care [42], which cannot be conclusively ascertained by prescription and referral rates.

Lastly, the symptoms of HF in our cohort may have been the GP’s highest priority to treat, as these symptoms have been shown to significantly reduce patients’ HRQOL [5, 43, 44]. In patients with advanced HF, the disease impairs the mental health scales of quality of life as much as major depression [5]. If patients are depressed because of their poor health status, the treatment of the original disease may be considered to be more important than the depression itself because the depression may improve as soon as the health status improves. To improve HRQOL in HF patients, treating HF symptoms may be as effective as the depression treatment.

#### Factors associated with depression treatment in heart failure patients

We hypothesised that GP awareness of depressive symptomatology was significantly associated with a higher utilisation of pharmacotherapy and psychotherapy. We found that HF patients whose GPs were aware of their depressive symptomatology (P(+/+) patients) had a significantly better chance to utilise pharmacotherapy (OR 2.8) than HF patients whose GPs were unaware of their depressive symptomatology (P(+/−) patients). However, we need to interpret these finding with some caution. As GPs had access to their patients’ reports, GPs could have used the treatment as a marker for the statement of a patient having shown depressive symptomatology. The link between GPs’ awareness of depressive symptomatology and psychotherapy was significant in bivariate analysis but lost significance when controlled for confounding factors. Instead, solely older age was significantly associated with a lesser chance (OR 0.9) of receiving psychotherapy. The significant association with age was consistent with prior research [14, 45, 46] and explained why there were only few cases of this elderly cohort receiving psychotherapy.

These (non-)associations between depression treatment utilisation and GP’s awareness of depressive symptomatology reflect the fact that pharmacotherapy can be prescribed by GPs themselves. Psychotherapy does not necessarily require the GP’s awareness of depressive symptomatology, nor does the GP’s referral to a psychotherapist suffice for a patient to receive psychotherapy. Due to the restricted number of therapy places, the barriers to psychotherapy are higher than to pharmacotherapy. Therefore, a GP’s influence on whether or not a patient receives psychotherapy (even though the GP may be aware of the depressive symptomatology and may recommend it) is limited.

#### Factors associated with health-related quality of life of heart failure patients with depressive symptomatology

HRQOL of all HF patients with depressive symptomatology (P(+) patients) at a mean age of 72.8 years scored 40.5 of 100 points on the EQ-5D VAS Scale. Compared to a 10-year older primary care cohort (65.4 points) [47] and a multimorbid primary care cohort at a similar age (62.4 points) [48], this was a comparably low HRQOL. Studies investigating HF patients found mean EQ-5D VAS scores between 54 and 62.6 points [49–51]). Therefore, besides HF, comorbid depression seems to considerably reduce HRQOL in our study. This impact of depression had been proven in earlier studies [52] and has also been confirmed by our multivariable analysis.

We hypothesised that GPs’ awareness of depressive symptomatology was significantly associated with higher HRQOL. Even though, the bivariate analysis showed that HRQOL significantly differed between patients whose GPs were aware of their patients’ depressive symptomatology (38.2 of 100 points) and patients whose GPs were unaware (41.7 points), the effect was small and opposed to the objective hypothesised. When controlled for confounders, neither the GP’s awareness of depressive symptomatology, nor the depression treatment, nor the interaction of both was significantly linked to HRQOL. Besides the severity of depression, patients’ NYHA Class was strongly associated with their HRQOL. This correlation to disease severity had also been shown in other studies [5, 50–52]. The lack of association of both, awareness of depressive symptomatology and depression treatment with HRQOL means that the depression treatment after getting aware of the depressive symptomatology did not suffice to improve HRQOL. Or the reasons might be more complex. The authors hypothesise that the severity of HF symptoms overruled the importance of a GP’s awareness of depressive symptomatology in regards to HRQOL in HF patients. Moreover, the GP’s awareness of depressive symptomatology might not have been associated with HRQOL because of the following reasons: Patients, whose depressive symptomatology the GP was aware of, might have been more affected by depression despite adequate treatment. At the same time, patients whose GP was unaware of their depressive symptomatology might have been less impaired although they had not received treatment. As a result, both groups did not significantly differ in terms of HRQOL. In other words: The GP was aware of the patients who were impaired by depressive symptoms and overlooked those who were less impaired. This hypothesis was supported by the fact that patients whose GP was unaware of their depressive symptomatology did in fact report a slightly higher HRQOL than patients whose GP was aware of their depressive symptomatology. This was true for both groups of patients (those with depression treatment and those without) as displayed in Table 8.

Overall, the authors did not find that GPs’ awareness of depressive symptomatology was significantly associated with patients’ HRQOL in contrast to the NYHA Class. Therefore, treating HF symptoms might be more important in improving the HRQOL in HF patients than systematically screening for depression in primary care. In earlier research, certain studies showed similar results and confirmed a positive link between depression treatment and depressive symptomatology [53]. They, however, failed to show a link between depression treatment and cardiac outcome in patients with cardiovascular diseases [53, 54]. Still, the need for depression awareness and treatment remains indisputable to reduce symptoms of depression in HF patients, independent of the possibility of improving HRQOL. We just need to differentiate  both outcome criteria.

#### Implications for research

There are several aspects which need to be considered in future research. Firstly, investigating the treatment of depression in primary care HF patients is more complex than it seems. Treatment options chosen by the GP (eg, dialogue with the patient) are not always hard outcome criteria for research but might be crucial in everyday practice. Outcome criteria need therefore be chosen carefully and qualitative studies may be helpful in regards to depression treatment in HF patients. Secondly, we need to keep in mind that studies focusing on patient utilisation behaviour do not necessarily reflect GPs’ efforts to provide treatment. Therefore, future research needs to differentiate between GP efforts to initiate treatment and patients’ acceptance and realisation of therapy options, especially in studies based on the claims data of health insurances. Factors leading to patients deciding not to use the recommended therapy options need to be investigated before deciding on the appropriateness of depression treatment in HF patients. Thirdly, the true dimension of a possible treatment gap needs to be further investigated, especially in those patients whose GPs are unaware of their patients’ depressive symptomatology. In the present study, the authors have found no significant link between GPs’ awareness of a depressive symptomatology and the patients’ quality of life. Therefore, one hypothesis may be that GPs are aware of relevant cases of depression in need of treatment and are unaware of less impaired patients without the need for treatment. However, a possible treatment gap should be investigated in longitudinal studies keeping the context of this hypothesis in mind.

Fourthly and last, the condition of HF may have such an impact on patients’ HRQOL that depression treatment (which is not significantly linked to HRQOL in the present study) may improve the symptoms of depression but not necessarily HRQOL as long as HF continues to significantly impair patients’ HRQOL. If this is the case, the recommendation to screen HF patients for depression and consequently treat depression will not improve patients’ HRQOL. Instead, measures beyond standard therapy of HF and depression may be necessary to further improve patients’ HRQOL. Especially questions as to why depression treatment does not improve HRQOL in patients with HF and what types of measures beyond standard therapy are necessary to do so should be further investigated. These aspects need to be considered when planning intervention studies in patients with HF and depression.

#### Implications for practice

GPs should be aware of the gap between GP-initiated and patient-utilised depression treatments in patients with chronic HF, which may lead to a undersupply of depression treatment, even though the GP has initiated treatment. Due to possible interaction effects between antidepressants and HF, close monitoring at the onset of taking antidepressants may improve patient adherence. Furthermore, GPs should critically consider the potentially general reluctance of referring older patients to psychotherapy. Additionally, there are probably patients who display depressive symptomatology and do not receive treatment because their GPs are unaware of the depressive symptomatology. GPs should keep in mind that depressive symptomatology is common in HF patients and, consequently, should pay special attention to HF patients’ potentially depressive symptomatology in order to avoid an undersupply of depression treatment. Due to the potentially harmful cardiac sideeffects of antidepressants (eg, described by *Rustard* et al. [41]) and the actual efforts to reduce polypharmacy, the type of treatment should be chosen carefully whilst considering non-pharmacologic treatment strategies for depression; exercise [55], cognitive behavioural therapy [22], and collaborative care [56] are beneficial in improving depressive symptoms in HF patients.

In general, we have to keep in mind that, just like the severity of HF, comorbid depression seems to considerably reduce CFH patients’ HRQOL. Therefore, if they have not already done so, GPs have to discuss with their patients the types of measures available to improve their HRQOL in addition to standard HF and depression therapy.

## Conclusion

If GPs knew of their patients’ depressive symptomatology, there were various GP-initiated treatments of depressive symptomatology in HF patients far exceeding pharmacotherapy or referrals to psychotherapists. Those data are not necessarily represented in routine data. In general practice, GPs should be aware of the gap between GP-initiated and patient-utilised depression treatments in patients with chronic HF, which might lead to a undersupply of depression treatment. Patients whose GP was unaware of their depressive symptomatology had a lower chance of receiving antidepressants, but surprisingly neither the GP’s awareness of depressive symptomatology nor depression treatment was significantly associated with the patients’ HRQOL. The authors hypothesise, that GPs might have been aware of HF patients with more severe depressive symptomatology resulting in a reduced HRQOL. GPs might have overseen depressive symptomatology without significant impairment of the patients’ HRQOL. Therefore, in the end, both groups of patients might have displayed comparable levels of HRQOL; the former patients with ongoing depression treatment and the latter without. This could explain the missing association between GPs’ awareness of depressive symptomatology and their patients’ HRQOL. This hypothesis will have to be further investigated in qualitative as well as longitudinal studies.

## Abbreviations

GP: General Practitioner
HF: Chronic heart failure
HRQOL: Health-related quality of life
